# Codon pair optimization (CPO): a software tool for synthetic gene design based on codon pair bias to improve the expression of recombinant proteins in *Pichia pastoris*

**DOI:** 10.1186/s12934-021-01696-y

**Published:** 2021-11-04

**Authors:** Yide Huang, Ting Lin, Lingfang Lu, Fan Cai, Jie Lin, Yu′e Jiang, Yao Lin

**Affiliations:** 1 Engineering Research Center of Industrial Microbiology, College of Life Sciences, Fujian Normal University, Fuzhou, 350007 China; 2 College of Mathematics and Informatics, Fujian Normal University, Fuzhou, 350007 China; 3 Provincial University Key Laboratory of Cellular Stress Response and Metabolic Regulation, College of Life Sciences, Fujian Normal University, Fuzhou, 350007 China; 4 Provincial University Key Laboratory of Sport and Health Science, School of Physical Education and Sport Sciences, Fujian Normal University, Fuzhou, 350007 China

**Keywords:** *Pichia pastoris*, Codon pair bias, Codon pair optimization, Synthetic gene design

## Abstract

**Background:**

Codon optimization is a common method to improve protein expression levels in *Pichia pastoris* and the current strategy is to replace rare codons with preferred codons to match the codon usage bias. However, codon-pair contexts have a profound effect on translation efficiency by influencing both translational elongation rates and accuracy. Until now, it remains untested whether optimized genes based on codon pair bias results in higher protein expression levels compared to codon usage bias.

**Results:**

In this study, an algorithm based on dynamic programming was introduced to develop codon pair optimization (CPO) which is a software tool to provide simple and efficient codon pair optimization for synthetic gene design in *Pichia pastoris*. Two reporters (MT1-MMP E2C6 and ADAM17 A9B8 scFvs) were employed to test the effects of codon pair bias and CPO optimization on their protein expression levels. Four variants of MT1-MMP E2C6 and ADAM17 A9B8 for each were generated, one variant with the best codon-pair context, one with the worst codon-pair context, one with unbiased codon-pair context, and another optimized based on codon usage. The expression levels of variants with the worst codon-pair context were almost undetectable by Western blot and the variants with the best codon-pair context were expressed well. The expression levels on MT1-MMP E2C6 and ADAM17 A9B8 were more than five times and seven times higher in the optimized sequences based on codon-pair context compared to that based on codon usage, respectively. The results indicated that the codon-pair context-based codon optimization is more effective in enhancing expression of protein in *Pichia pastoris*.

**Conclusions:**

Codon-pair context plays an important role on the protein expression in *Pichia pastoris*. The codon pair optimization (CPO) software developed in this study efficiently improved the protein expression levels of exogenous genes in *Pichia pastoris*, suggesting gene design based on codon pair bias is an alternative strategy for high expression of recombinant proteins in *Pichia pastoris*.

**Supplementary Information:**

The online version contains supplementary material available at 10.1186/s12934-021-01696-y.

## Background

There are 64 codons in genetic codon dictionary, but the number of amino acids composed of proteins is only 20 in nature. On total, 18 amino acids are encoded by two to six synonymous codons except for methionine and tryptophan. However, synonymous codons in protein coding sequences are not used with equal frequencies by organisms in gene evolution [1–4]. This phenomenon is known as codon usage bias (CUB). Therefore, the codons in genes not only determine the amino acid sequences, but also are one of the important determinants for translational efficiency. Studies showed that not only the usage of synonymous codons has preference, but also the contexts of codons have certain patterns in protein coding sequences. The 5′ codon is juxtaposed non-randomly with 3′ codon, this phenomenon is typically referred to as codon pair bias (CPB) or codon context bias [5–7]. It is generally believed that codon context influences translational elongation rates and accuracy, by the compatibilities of adjacent tRNA molecules in the P- and A-sites on the surface of the large subunits of translating ribosomes [8–11]. Studies showed that codon context is more strongly related to translational efficiency than single codon usage [8, 12, 13]. However, CPB has been relatively ignored compared to CUB when considering for gene synthesis.

Codon optimization for transgenes is one of the most common approaches to improve the expression of recombinant proteins in host cells [14–16]. It is an easy way to optimize gene sequences based on CUB, which involves only the substitution of a single codon for each optimized codon. Gene sequence optimization based on CPB is to select the appropriate codon for the amino acid in the current position according to the optimal codon that the amino acid at the previous position may use, so as to optimize the context preference between the codon and the pre-codon. In this process, since the amino acid at each position have several synonymous codons, and there may be more than one synonymous codon for the optimal combination with the codons used by the pre-amino acid, therefore the codon coding combinations that constitute the specified amino acid will have $m_{1} \times m_{2} \cdots \times m_{i} \cdots \times m_{k}$, of which $m_{1}$ is the number of synonymous codons of the first amino acid, $m_{i}$ is the number of optimal synonymous codons of the i-th amino acid (which needs to be determined according to the amino acid codon at the previous position), K is the number of amino acids in the protein, and $1 \le i \le K$. The number of synonymous codons of most amino acids is greater than or equal to 2, and the combination number is at least $2^{k}$. When k increases, the number of candidate combinations increases exponentially. When k = 20, there are 1 million candidate combinations. Therefore, it is difficult to select the optimal combination manually.

*Pichia pastoris* (also known as the genus *Komagataella*), a methylotrophic yeast, has become an important industrial microorganism and has been widely employed in laboratories all over the world as cellular factory to produce recombinant proteins for basic research and medical applications since it was developed as a protein expression system in 1989 [17–19]. The genome of *Pichia pastoris* was firstly sequenced and assembled in 2009, and CPB was analyzed by the authors. Their analysis showed that some synonymous codon pairs are more or less frequently used than expected, indicating that the codon-pair context in *Pichia pastoris* is biased [20]. Until now, it remains untested in *Pichia pastoris* whether optimizing genes based on CPB results in higher protein expression levels. In this work, based on the data of amino acid synonymous codon context preference, The dynamic programming algorithm was used to decompose the above problem into an optimization problem between a protein sequence with the number of amino acids k-1 and an amino acid with a length of 1 firstly, then continue to decompose the protein sequence into an optimization problem between a sequence with length k-2 and an amino acid recursively. The optimization problem is decomposed into smaller problems recursively, and then combine the sub-problems from small to large as the solution of the final problem. Finally, the optimal solution can be obtained in linear time and the optimized sequence of codons can be obtained. The single chain antibody fragments (scFvs) were used as the reporters to evaluate the expression levels of optimizing sequences based on CPB in *Pichia pastoris*.

## Results and discussion

### Developing a software tool based on codon-pair context for efficient synthetic gene optimization in *Pichia pastoris*

Studies unveiled that codon-pair context is biased across species in the three domains of life [5, 6, 21–27]. After genome sequencing, codon-pair context of *Pichia pastoris* was analyzed [20]. From the codon-pair context map, *Pichia pastoris* shows a strong CPB and four codon-pair context rules can be easily found: the rejected codon-pairs are X1X2U3–A1Y2Y3 and X1X2C3–G1Y2Y3 and the favored codon-pairs are X1X2U3–G1Y2Y3 and X1X2C3–A1Y2Y3, where X and Y represents any base. The codon-pair context is related to the decoding speed and accuracy of mRNA, which affects the expression levels of proteins and critical for protein expression platforms such as *Pichia pastoris*. There are few studies suggested that optimization of heterologous gene expression based on codon context may be a better strategy for improving protein expression [10, 28, 29]. Single codon optimization according to CUB is based on the performance of the codons in proteins, which only consider the performance of the codon in the current position, but cannot optimize the adjacent codons according to the relationships of front and back position of the current codon. The pairwise codons optimization based on CPB not only takes into account the performance of codon combinations, but can find the globally optimal combination sequences of codons by optimizing the adjacent codons based on the front and back positions. It is necessary and meaningful to optimize pairwise codons by a suitable algorithm due to the high computational complexity caused by the simple combination method.

Because the number of candidate optimal combinations increases exponentially when simply using the gene sequence optimization method based on codon pairwise bias, an algorithm based on dynamic programming was introduced, which effectively solves the problem of exponential expansion of the number of candidate optimal combinations. The algorithm uses recursive idea to solve the problem of protein sequence optimization. The main idea is to decompose the problem to be solved into several sub-problems. This sub-problem is also the protein sequence optimization problem, but the input range is a subset of the original problem. The form of the sub-problem is the same as the original problem, but the scale of the problem is reduced, which makes it simpler and easier to solve. Then the sub-problems are further decomposed, and then decomposed into sub-problems of sub-problems, until the problem is small enough and easy to solve, and then the solution of each sub-problem is returned. In recursive calculation, the solution of each sub-problem provides useful information for the solution of the parent problem, and finally the optimal solution of the original problem is obtained.

**Fig. 1:**
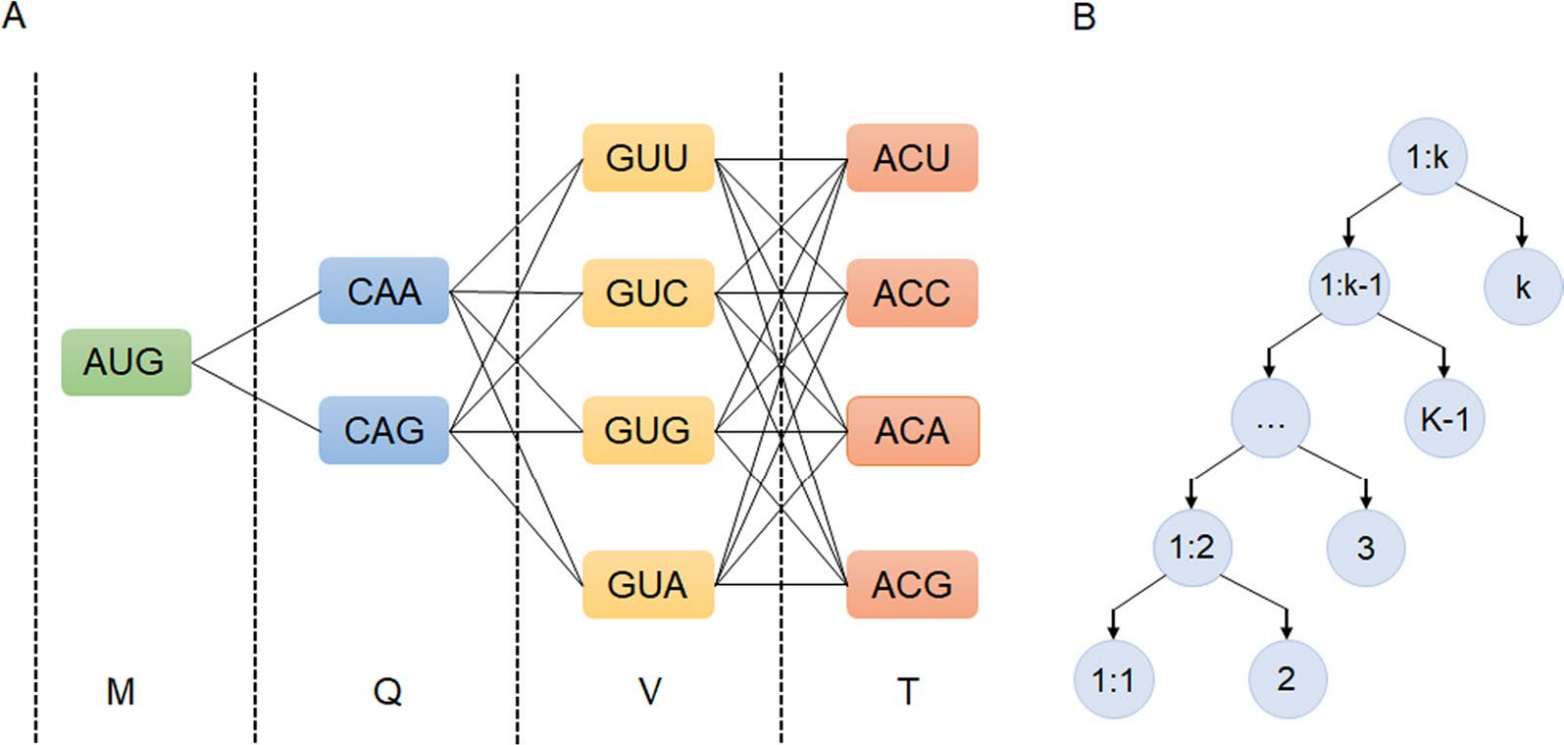
The sketch figures of the codon optimization algorithm. **A** The demo graph G of the sequence MQVT, M: Methionine, Q: Glutamine, V: Valine, T: Threonine. **B** The recursively decomposed tree

Firstly, define graph G, according to the number of amino acids in the protein sequence to be expressed. The G is divided into n layers, each layer corresponds to each amino acid in the sequence. The nodes of each layer are codons corresponding to amino acids, and the number of nodes in each layer is the number of codons corresponding to the amino acid. Each layer forms a fully connected subgraph. That is, each layer of nodes has an edge connected to the next layer of nodes. Assuming there are two nodes in this layer. The next layer has four nodes, and then there are 2 × 4 = 8 edges connected. Figure 1A showed the graph G of the sequence MQVT: Valine (Val, expressed in alphabetical V), which has four corresponding codons GUU, GUC, GUG and GUA. So, on the V floor, there are 4 nodes. Glutamine (Gln, in the previous layer expressed in alphabetical Q), which has two corresponding codons, CAA and CAG. So, at Q level there are two nodes. On total there are 2 × 4 = 8 edges between the two layers.

Secondly, the optimization problem is decomposed, and the protein sequence optimization problem with length k is divided into two sub-problems. One is the optimization problem of protein sequence with length k-1, the other is the problem of optimal solution between the remaining protein sequence of length 1 with the previous sub-problem. Then, the sub-problem of protein sequence optimization with length of k-1 is decomposed into K-2 optimization problem and optimization problem with the remaining protein sequence with length 1 recursively. As shown in Fig. 1B, the whole problem can be recursively decomposed into optimization problems between k protein sequences with length 1 and protein sequence optimization sub-problems with length K-1, K-2, K-3,…,2, 1. The form of sub-problems is the same or similar to the original problem, but the scale of the problem is reduced, which makes it simpler. Moreover, the algorithm ensures that each sub-problem is solved only once, and the solutions is stored in a two-dimensional array, and the query is carried out when necessary, which will not cause the problem of repeated calculations.

Thirdly, from bottom to top, the combinatorial optimization solutions of the sub-problems in the left and right sub-trees are calculated. The first kind of sub-problems of the next level and the combinatorial optimization problem of its two sub-nodes (i.e. the second sub-problem) can be solved by solving the first kind of sub-problems above, that is, solving the problems at the inner nodes of the tree. Therefore, the problem that needs to be solved is how to calculate the combinatorial optimization solution of the sub-problems of two sub-trees after decomposing the sequence. A group of values of each variable related to a sub-problem is called a “state”. A state corresponds to the value of one or more sub-problems in a certain state, which is the solution of the sub-problem corresponding to the state. The collection of all states is called “state space”, and the state space is the combination of states among related sub-problems.

The values used to calculate the optimization solution of the sub-problems were shown in Additional file 2: Table S1. Additional file 2: Table S1 represented the color-coded map of codon pair usage for *Pichia pastoris* described in the literature [20] that was converted into numbers (− 2, − 1, 0, 1 and 2). The negative numbers represent the rejected 3′ codon context, the positive numbers the preferred 3′ codon context and zero indicates no statistical significance.

Taking Fig. 1A as an example, suppose four optimized solutions (states) of MQV sub-sequence have been obtained, each of which is connected from the codon in M to the corresponding codon of V, since V corresponds to 4 codons. There are 4 optimized solutions, which end with the four codons corresponding to V. The problem that needs to be solved now is the optimized solution obtained after adding the codon of T amino acid to the MQV sub-sequence using the four optimized solutions. Amino acid T has four codon expressions (states), so four optimized solutions of MQV sub-sequence can be gotten. MQV already has four optimal paths, the optimal paths among each path arriving at the four codons of T one by one were solved, that is to add the four codons of V to each path one by one as the end point, and calculate the value of their reaching the end point. Therefore, in this example, 4 × 4 paths need to be calculated, from which the optimal solution to each end point is selected as the four states of MQVT sub-sequence.

It can be seen from the above example that the size of the state space of the sequence optimization problem is actually the sum of the product of the number of codons corresponding to each amino acid and the number of codons corresponding to the next amino acid, which can be written as the following equation:

$$S = \sum\nolimits_{i = 1}^{k - 1} {\left| {r_{i} } \right|} \times \left| {r_{i + 1} } \right|,$$

where S is the size of the state space of the problem, K is the number of amino acids in the protein sequence to be optimized, |r_i_| is the number of codons corresponding to the i-th amino acid.

Finally, the optimal path from the first amino acid to the last amino acid is obtained through the above steps, and the maximum value is selected as the optimal solution for output.

### Effect of codon-pair context on the expression of proteins in *Pichia pastoris*

**Fig. 2:**
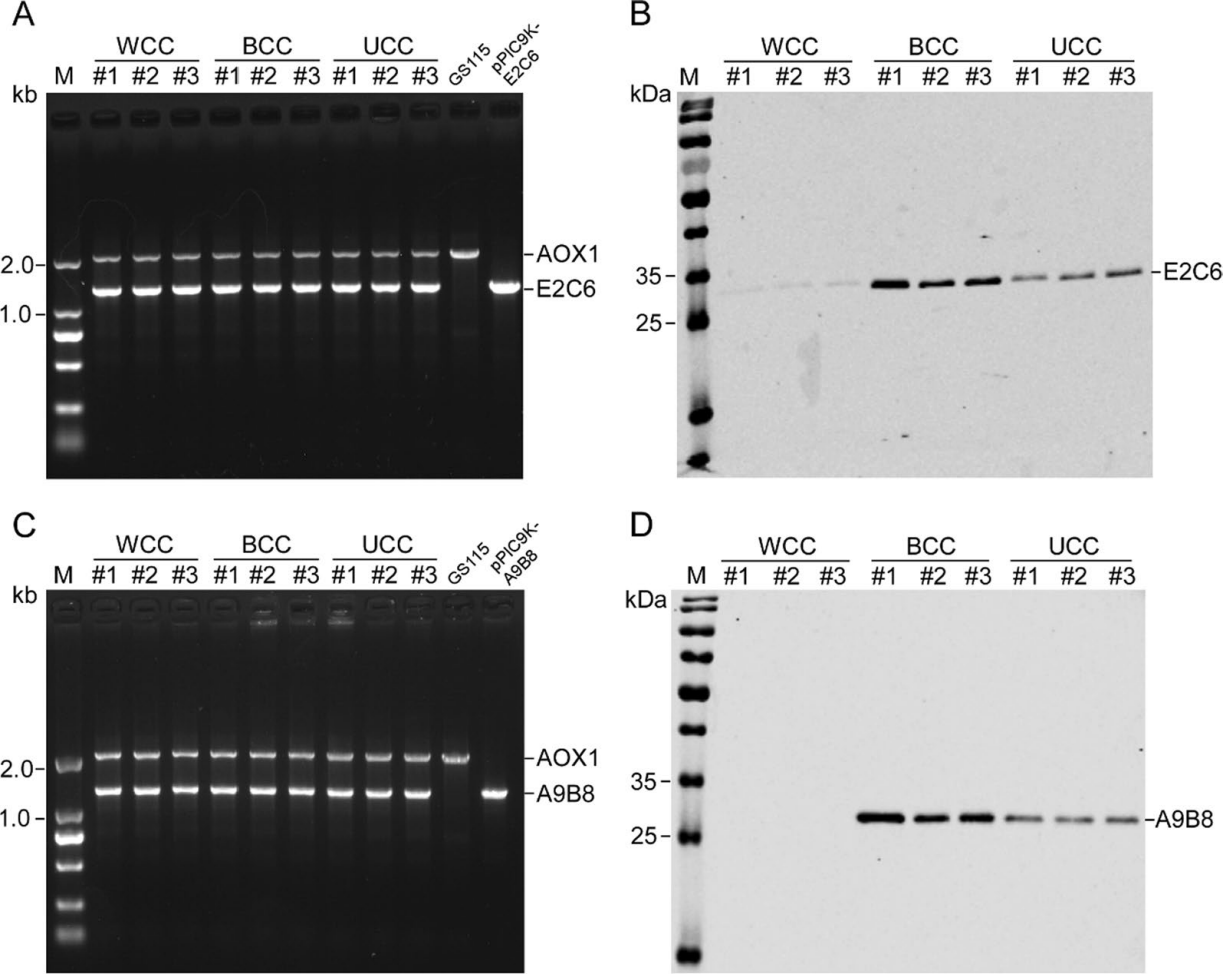
The expression of variants of MT1-MMP E2C6 and ADAM17 A9B8 with the worst, the best, and the unbiased codon-pair context in *Pichia pastoris*. **A**, **C** Analysis of integrants using *AOX1* general primers by PCR. Lane GS115 is a control using the host GS115 genome as the template, which show AOX1 gene, and lane pPIC9K-E2C6 or pPIC9K-A9B8 is another control using recombinant plasmid pPIC9K-E2C6 or pPIC9K-A9B8 as the template, which shows synthetic gene of MT1-MMP E2C6 and ADAM17 A9B8. **B**, **D** The expression of variants in *Pichia pastoris*. MT1-MMP E2C6 and ADAM17 A9B8 in the supernatant are detected by Western blot using anti-his tag primary antibody. #1, #2 and #3 represent three different recombinant strains. *WCC* the worst codon-pair context, *BCC* the best codon-pair context, *UCC* the unbiased codon-pair context

To investigate the effect of codon-pair context on the expression of proteins in *Pichia pastoris*, two single chain antibody fragments, MT1-MMP E2C6 and ADAM17 A9B8, were employed as the reporters. Three variants of MT1-MMP E2C6 and ADAM17 A9B8 for each were generated, one variant with the best codon-pair context, one with the worst codon-pair context, and another with the unbiased codon-pair context. Each variant was inserted into the yeast expression vector of pPIC9K and transformed into *Pichia pastoris* GS115. Three colonies grown on MD screening plates for each were selected and analyzed by PCR to further confirm that the insertions were successfully integrated into the genome of *Pichia pastoris* (Fig. 2A, C). The growth of cells was monitored by OD_600_ at time points (0, 24, 48 and 72 h) and the total protein concentration of the supernatants was determined by BCA assay. Both the cell growth and total proteins were not significantly different in the strains expressing MT1-MMP E2C6 or ADAM17 A9B8 (Additional file 1: Fig. S1). The MT1-MMP E2C6 and ADAM17 A9B8 in the supernatant of fermentation were detected using anti-His tag antibody. The results were observed that the variant of MT1-MMP E2C6 with the worst codon-pair context was weakly expressed and the expression of ADAM17 A9B8 variant with the worst codon-pair context was undetectable, while the variants of both MT1-MMP E2C6 and ADAM17 A9B8 with the best codon-pair context were expressed well. The variants of both MT1-MMP E2C6 and ADAM17 A9B8 with the unbiased codon-pair context were also expressed, but their expression levels were lower than that of the variants with the best codon-pair context (Fig. 2B, D). These results demonstrated that codon-pair context plays an important role on the protein expression in *Pichia pastoris*.

### The sequences optimized using codon-pair context showed better expression of proteins than that using codon usage

**Fig. 3:**
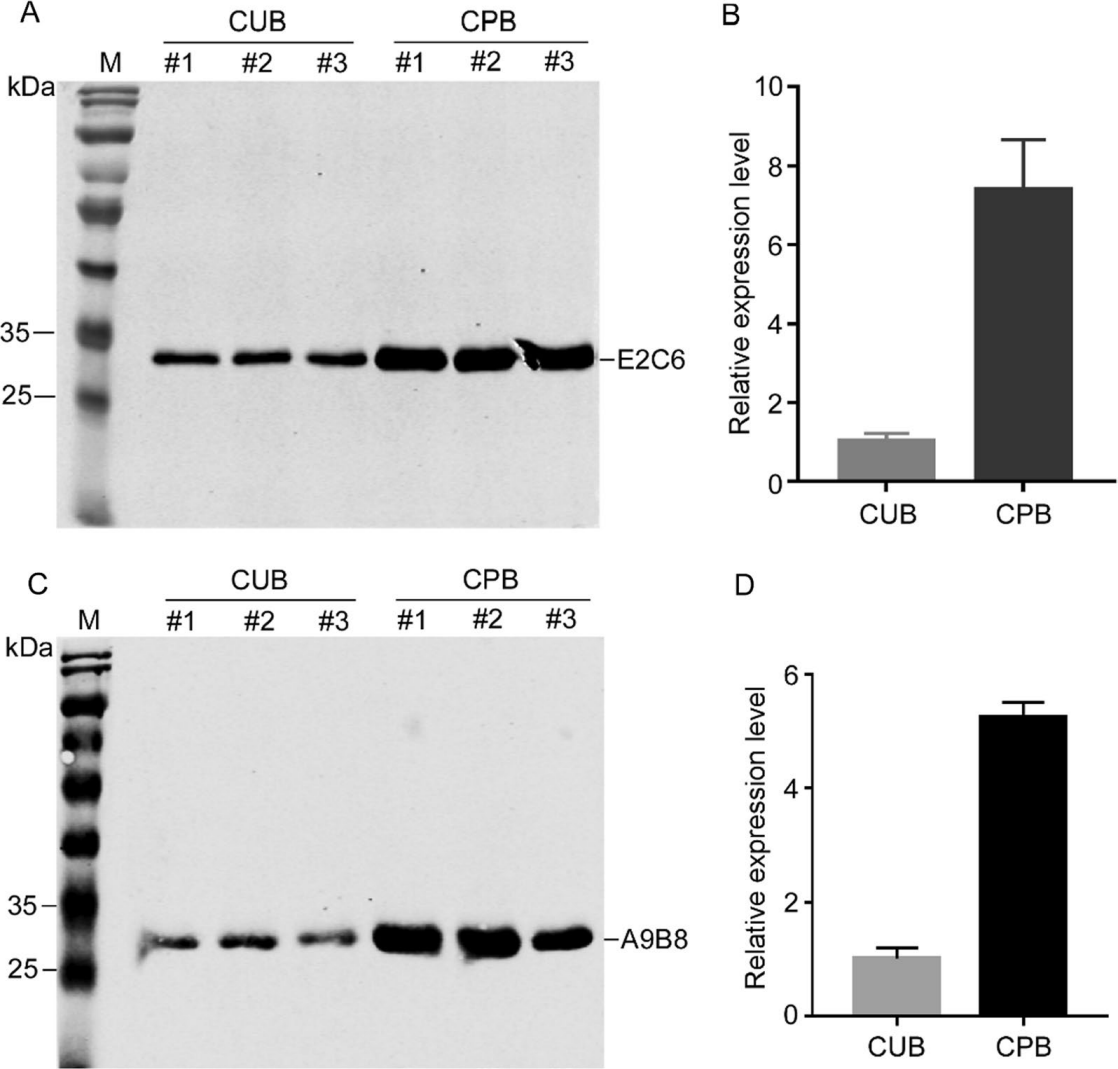
The expression of the optimized sequences designed with codon usage and codon-pair context in *Pichia pastoris*. **A**, **C** The expression analysis of MT1-MMP E2C6 and ADAM17 A9B8 by Western blot using anti-his tag primary antibody. **B**, **D** The quantitative analysis to **A**, **C**. #1, #2 and #3 represent three different recombinant strains. *M* protein marker, *CUB* codon usage bias, *CPB* codon pair bias

The codon optimization of foreign genes is a widely used method to improve the protein expression levels in host organism. The common strategy for codon optimization is to replace rare codons with preferred codons to match the CUB of the host organism, which is also widely used in *Pichia pastoris *[14, 30, 31]. Can the optimized sequences produced from codon-pair context perform better in protein expression than that produced from codon usage in *Pichia pastoris*? To answer the question, the optimized sequences of MT1-MMP E2C6 and ADAM17 A9B8 based on codon usage and codon-pair context were expressed in *Pichia pastoris* and the expression levels of MT1-MMP E2C6 and ADAM17 A9B8 in the supernatant of fermentation were evaluated by western blot. The results showed that the expression levels on MT1-MMP E2C6 and ADAM17 A9B8 were more than five times and seven times higher in the optimized sequences based on codon-pair context compared to that based on codon usage, respectively (Fig. 3). These results suggested that codon-pair context could be more important for protein expression than codon usage, and CPO works as expected and the optimization based on CPO efficiently influences the protein expression levels in *Pichia pastoris*.

In 2012, Chung et al. evaluated the relative importance of optimizing individual codon usage and codon-pair context for enhancing protein expression in *Escherichia coli*, *Lactococcus lactis*, *Pichia pastoris* and *Saccharomyces cerevisiae* [32]. Their results suggested that codon-pair context is a more relevant design criterion than individual codon usage [32]. Researches using reporter proteins also showed that the optimized sequences based on CPB increased more protein production than that based on CUB in *Pichia pastoris* and CHO expression systems [29, 33]. At present, the database of genomic codon-pair statistics of all organisms with sequenced genome in the GenBank has been constructed [34]. Combination of codon-pair usage data in the database and algorithm developed in this study will be easy to optimize genes based on CPB in many organisms. Of note, most studies only compared CPB versus CUB using reporter proteins. Native proteins may react other ways compared to reporter proteins.

**Fig. 4:**
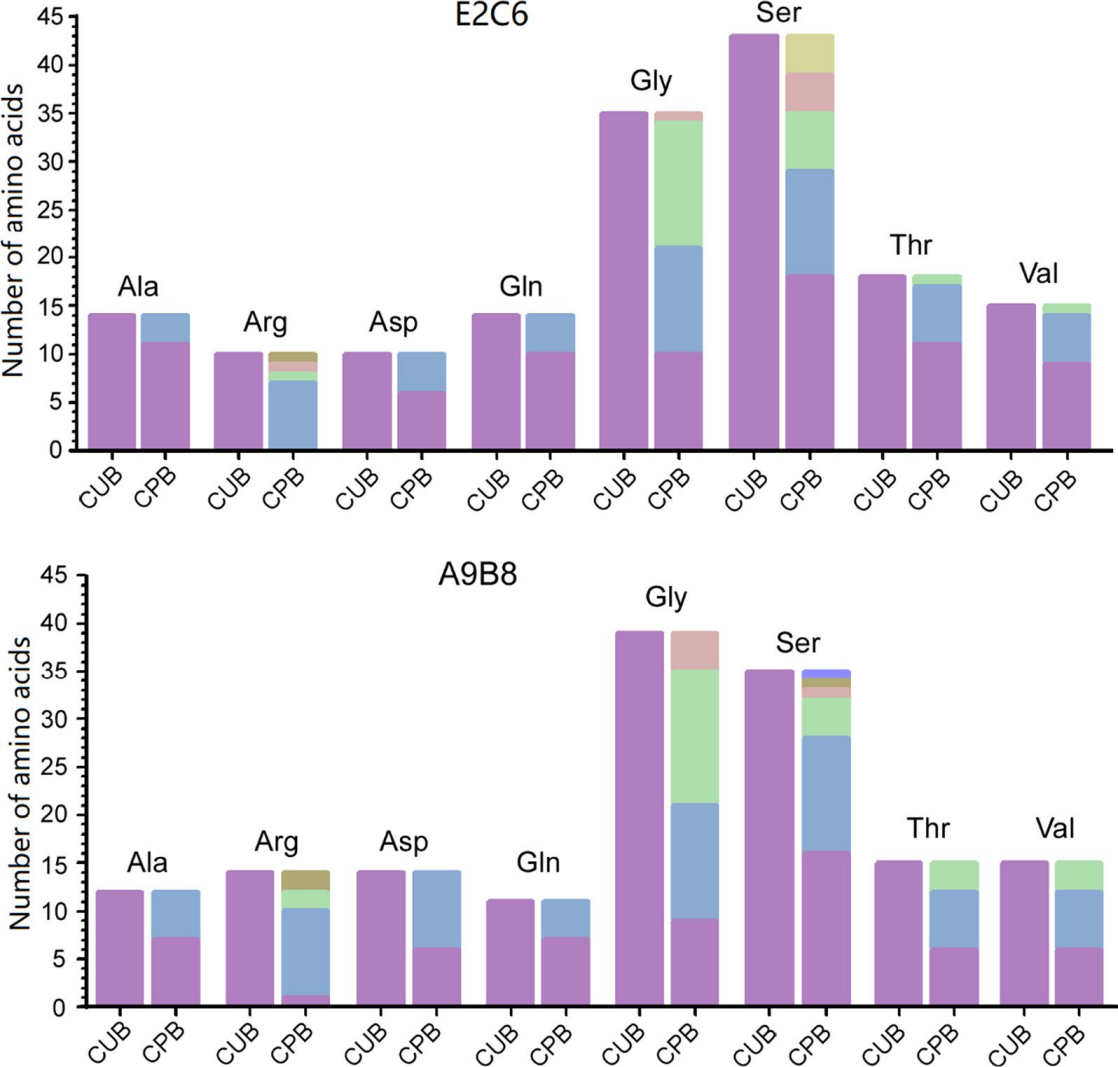
The usage of synonymous codons in the optimized sequences. The type and number of amino acids appearing no less than 10 times in the optimized sequences were listed. Different colors in the column represent different synonymous codons of the specific amino acids. *CUB* codon usage bias, *CPB* codon pair bias

**Fig. 5:**
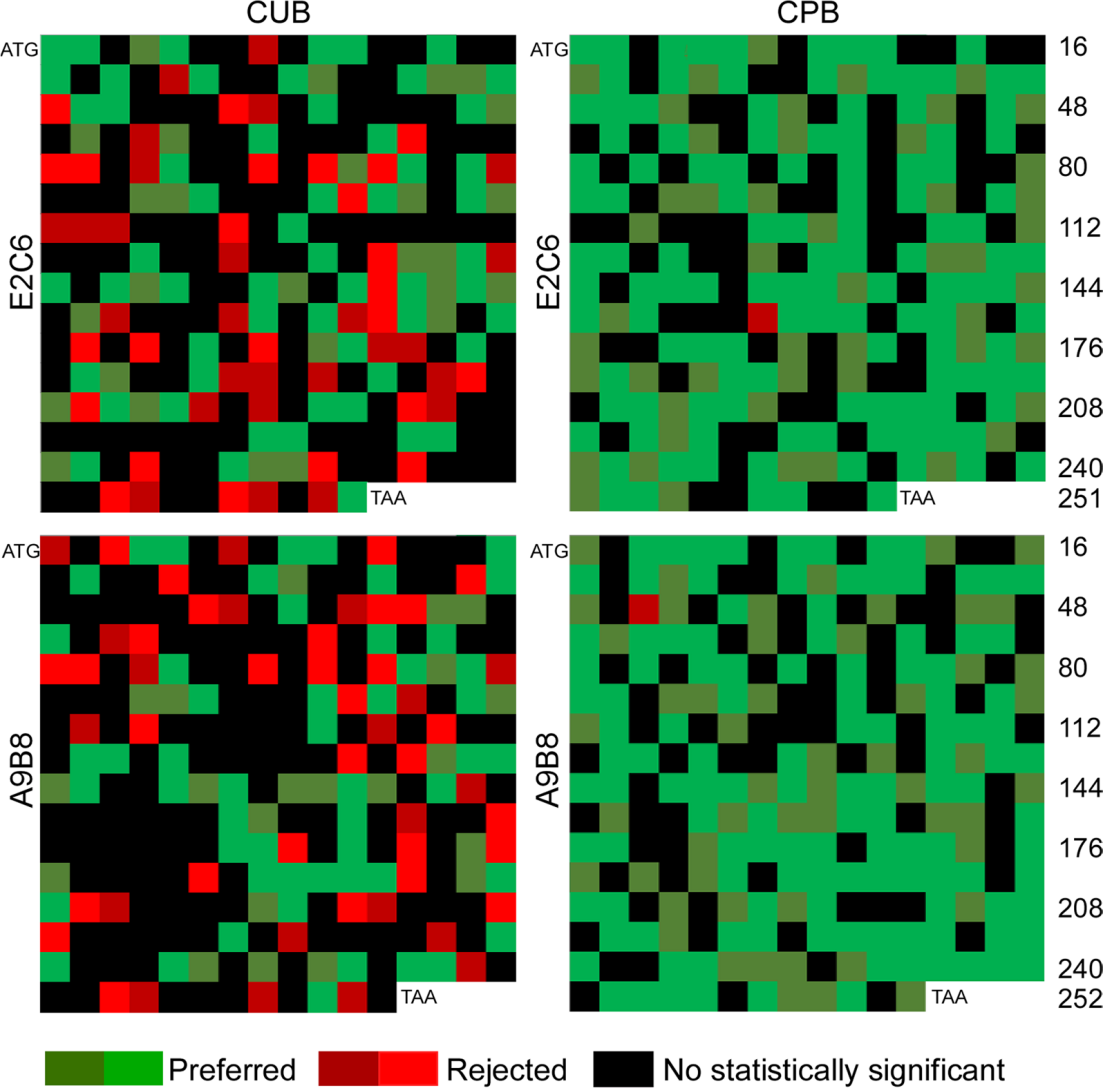
The codon-pair context map of optimized sequences of MT1-MMP E2C6 and ADAM17 A9B8. In order to better demonstrate codon-pair context of optimized sequences of MT1-MMP E2C6 and ADAM17 A9B8 based on CUB and CPB, the codons of optimized sequences were arranged into the map with 16 × 16 cells from left to right in sequence. Each cell in the map corresponds to a codon encoding a specific amino acid. The start codon ATG is displayed in the upper left corner of map and the stop codon TAA in the bottom right corner of map and the number of codons on the right of maps. The codon in the cell with its 5′ neighbor codon was colored in red for rejected contexts, green for preferred ones and black for ones without statistical significance. The intensity of red and green indicates the extent of the preference or rejection. *CUB* codon usage bias, *CPB* codon pair bias

The usage of synonymous codons in the optimized sequences from codon usage and codon-pair context was further analyzed. First, the usage of synonymous codons is very different for frequent amino acids (appear no less than 10 times) in the optimized sequences of MT1-MMP E2C6 and ADAM17 A9B8. In the sequence based on CUB, same codon is always selected for a specific amino acid, yet in the sequence based on CPB, the codons employed varied (Fig. 4). A possible mechanism underlying this observation may be the limit of translation speed by the number of the charged tRNA in a cell. For example, there are 39 Glycines and 35 Serines in ADAM17 A9B8, and 35 Glycines and 43 Serines in MT1-MMP E2C6 sequences. Various codons rather than a single codon were selected for these amino acids in the sequence based on CPB in comparison to the sequence based on CUB, which greatly relieved the lack of charged tRNA number in cells to satisfy the translation machinery. Second, the codon-pair context was also analyzed in the optimized sequences based on CUB and CPB. For both MT1-MMP E2C6 and ADAM17 A9B8 sequences, there are 50 rejected codons in the optimizations based on CUB, yet there are only one in those based on CPB (Fig. 5). These rejected codon-pair contexts in both optimized sequences of MT1-MMP E2C6 and ADAM17 A9B8 based on CUB may be another barrier to decrease the expression levels of MT1-MMP E2C6 and ADAM17 A9B8 in *Pichia pastoris*.

## Conclusions

In this work, codon pair optimization (CPO), a software tool to provide codon pair optimization for synthetic gene design, was developed. CPO provides a simple and efficient means for customizing codon optimization based on codon pair bias of *Pichia pastoris*. The optimization based on CPB using CPO demonstrated a significantly higher protein expression level in *Pichia pastoris* compared to the traditional optimization based on CUB, suggesting CPO provides a useful tool of effective optimization for high synthetic gene expression in *Pichia pastoris*.

## Materials and methods

### Plasmid and strains

Plasmid pPIC9K was used as the cloning and protein expression vector. The expression of the reporters was carried out in *Pichia pastoris* strain GS115 and *Escherichia coli* Top10′ was used as the cloning host cell for genetic manipulation.

### Media and growth conditions

Luria–Bertani (LB) medium (0.5% yeast extract, 1% peptone, and 0.5% NaCl) supplemented with 100 μg/ml Ampicillin was used to culture bacterial strains, and bacteria were grown at 37 °C in a heated incubator or orbital shaker. Yeast strains were cultured in yeast peptone dextrose (YPD) medium (1% yeast extract, 2% peptone, 2% dextrose), and MD plates (2% dextrose, 1.34% yeast nitrogen base, 400 μg/l biotin and 2% agar) to screen transformants. To express the reporters, recombinant strains were cultured in buffered complex glycerol medium (BMGY) (1% yeast extract, 2% peptone, 0.34% YNB, 4 × 10^–5^% biotin, 0.5% methanol, 100 mM potassium phosphate, pH 6.0) and buffered complex methanol medium (BMMY) (1%yeast extract, 2% peptone, 100 mM phosphate buffer, pH 6.0, 1.34% yeast nitrogen base, 4 × 10^–5^% biotin, and 1% methanol). All yeast strains were grown at 30 °C in an incubator or orbital shaker.

### Preparation of synthetic genes and plasmids

A total of eight sequences of MT1-MMP E2C6 [35] and ADAM17 A9B8 [36] scFvs were generated according to CUB and CPB of *Pichia pastoris*, two from CUB (here named as CUB-E2C6 and CUB-A9B8), two from CPB with the highest score (here named as BCC-E2C6 and BCC-A9B8), two from CPB with the lowest score (here named as WCC-E2C6 and WCC-A9B8), and two with unbiased codon-pair context as references. CUB-E2C6 and CUB-A9B8 were optimized according to the codon usage table of *Pichia pastoris* reported by Schutter et al. [20]. BCC-E2C6, BCC-A9B8, WCC-E2C6 and WCC-A9B8 were designed using the software tool described in this study. To facilitate protein detection by Western blot, the sequence encoding 6× His tag was inserted upstream to the synthetic genes. The all eight sequences were synthesized by Wuhan GeneCreate Biological Engineering Co., Ltd, and subcloned into plasmid pPIC9K in frame and downstream of the α-factor signal sequence at SnaBI site.

### Electroporation of *Pichia pastoris*

The electroporation of *Pichia pastoris* was described in detail in Huang et al. [37]. The recombinant plasmids were linearized with SalI restriction endonuclease. 10 μg linearized plasmid DNAs were transformed into the yeast strain GS115 by electroporation (1.5 kV, 25 µF, 200 Ω) using a Pulser (Bio-Rad, Hercules, USA). The transformed cells were grown on MD plates at 30 °C for 2–3 days until colonies appeared.

### PCR analysis of integrants

PCR was employed to determine if the genes of MT1-MMP E2C6 and ADAM17 A9B8 scFvs have integrated into the genome of the strain GS115. The genomic DNAs from clones grown on MD plates were isolated according to the Pichia Manual Protocol. Amplification of the gene of interest was carried out with the 5′ *AOX1* forward primer (GACTGGTTCCAATTGACAAGC) and the 3′ *AOX1* forward primer (GGCAAATGGCATTCTGACATCCTC) using 200 ng of genomic DNA as template. The PCR procedure was set as 94 °C for 5 min for one cycle, 30 cycles of 94 °C for 30 s, 55 °C for 20 s and 72 °C for 1 min. The PCR products were analyzed by 1% agarose gel.

### Expression of protein

The transformants confirmed by genomic PCR assay were firstly cultured in BMGY medium in 100 ml glass flasks in a maximum volume of 20 ml at 220 rpm for 24 h until the OD_600_ reached 2–5. The cells were pelleted at 1500×*g* for 10 min at room temperature and resuspended to an OD_600_ of 1.0 (about 5 × 10^7^ cells/ml) in BMMY medium and cultured in 250 ml glass flasks with a maximum volume of 25 ml. Then 25 μl of 100% Methanol was added to induce protein expression at 24 h intervals and the growth of cells was monitored from the OD_600_ value every 24 h. After shaking in flasks for 72 h, the supernatant was harvested by centrifugation at 1500×*g* for 10 min at 4 °C. Because the expression levels of proteins were determined based on the biomasses [29], the OD_600_ of each strain was checked again and the fermentation broth was balanced with BMMY medium according to the value of OD_600_ to ensure the same number of cells per ml of broth in each strain before harvesting the supernatant by centrifugation. The total proteins of the supernatant were determined by BCA assay using BSA as the standard protein.

### Western blot

The expression of reporters was analyzed by Western blot. Briefly, 10 μl of supernatant was separated on 12% SDS-PAGE. After transferring the proteins in gel to Hybond-C nitrocellulose membrane (Amersham Bioscience, Little Chalfont, UK), the NC membranes were blocked with 5% fat-free milk and incubated overnight at 4 °C with anti-His tag antibody (Sangon Biotech, China, Cat no. D191001). IRDye 800CW-conjugated goat-anti-Rabbit secondary antibodies (LI-COR Biosciences, Lincoln, NE, USA; cat. no. C60607-15) was employed to detect the hybridization signal at 1:1000 dilutions. The signals were measured using LICOR Odyssey system (LI-COR, Nebraska, USA).

## Supplementary Information

**Additional file 1: Figure S1.** Cell growth and total protein concentration in the supernatants.

**Additional file 2: Table S1.** Codon-pair context values used to calculate the optimization solution in CPO software.

## Data Availability

All data generated or analyzed during this study are included in this published article and its additional files.
