# Supplementary material for: Codon pair optimization (CPO): a software tool for synthetic gene design based on codon pair bias to improve the expression of recombinant proteins in Pichia pastoris
Source: Microb Cell Fact. 2021 Nov 4;20:209. doi: 10.1186/s12934-021-01696-y (PMC8567542; doi:10.1186/s12934-021-01696-y)
Supplement: Supplementary file 1 — Additional file 1: Figure S1. Cell growth and total protein concentration in the supernatants. [file 12934_2021_1696_MOESM1_ESM.docx]

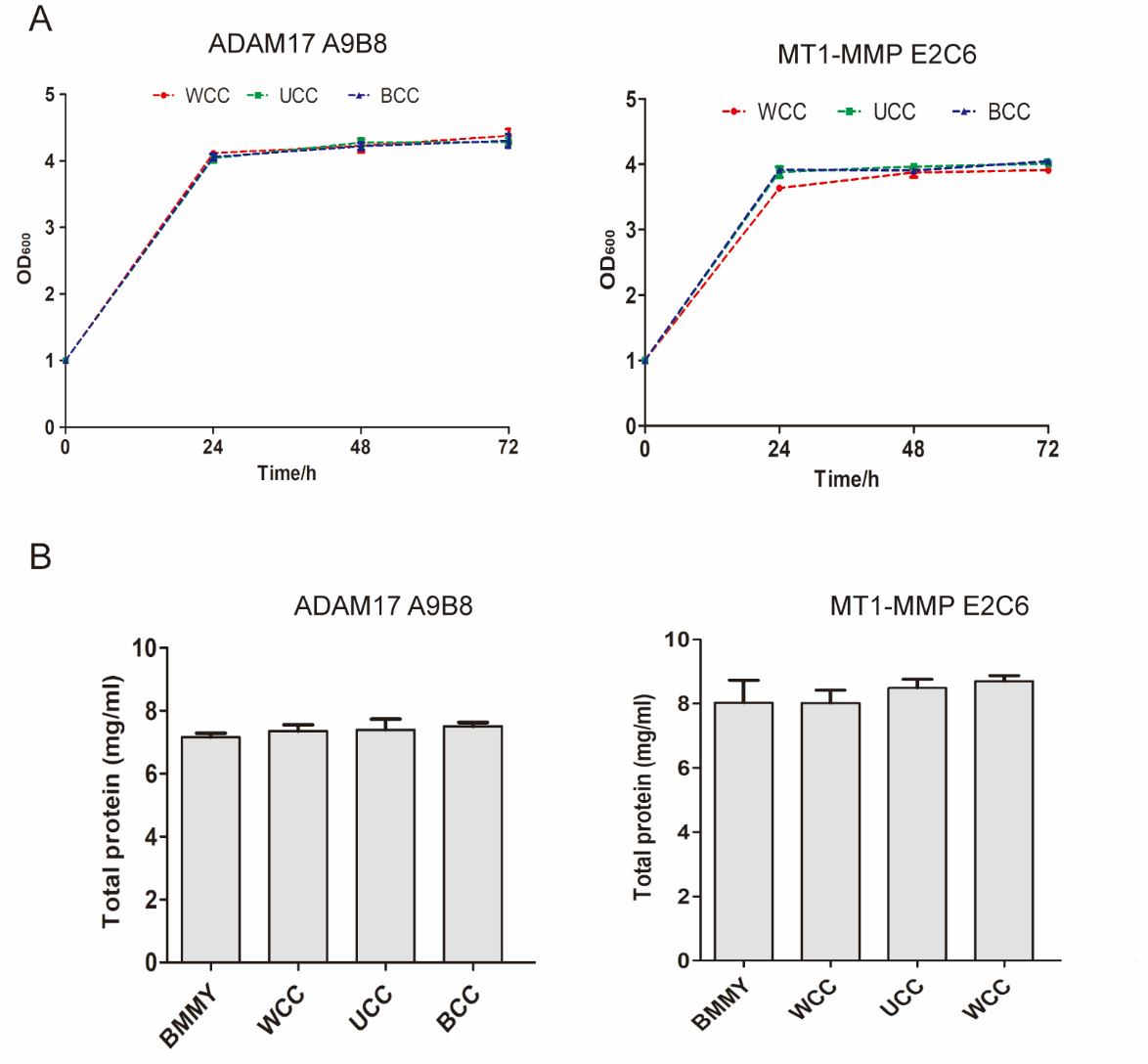


Figure S1. Cell growth and total protein concentration in the supernatants. A. The growth of cells expressing ADAM17 A9B8 and MT1-MMP E2C6. The OD600 was monitored at time points (0, 24, 48 and 72 h). B. The total protein concentration in the supernatants was determined by BCA assay after fermentation for 72 h. WCC, the worst codon-pair context; UCC, the unbiased codon-pair context; BCC, the best codon-pair context; BMMY, BMMY medium.
