# Supplementary material for: Codon pair optimization (CPO): a software tool for synthetic gene design based on codon pair bias to improve the expression of recombinant proteins in Pichia pastoris
Source: Microb Cell Fact. 2021 Nov 4;20:209. doi: 10.1186/s12934-021-01696-y (PMC8567542; doi:10.1186/s12934-021-01696-y)
Supplement: Supplementary file 2 — Additional file 2: Table S1. Codon-pair context values used to calculate the optimization solution in CPO software. [file 12934_2021_1696_MOESM2_ESM.zip › Readme.pdf]

The usage of the CPO package

1. Unzip the CPOpackage.zip into a directory.
2. Open the R and set the work directory to the directory.
3. Source the package.
4. Call the CPO function with the protein sequence.

```
> source("CPO.R") # Load the CPO package.
> pro="MVSTGVQPLDDCIQKFHDLKMKQTFRYIIYKIDGDNIVTDSVGNREETFENFIKVIPQNNARYAVFDYYFKTNEVPSREIQKLIFIFYCPDTAPLKEKMLYASSKDD" # The protein sequence.
> ret=CPO(pro)     # Call the CPO function.
> ret$codon.list  # Return the Optimizated codon sequence.
[1] "AUG-GUU-UCC-ACU-GGU-GUU-CAA-CCG-UUG-GAU-GAU-UGC-AUU-CAA-AAG-UUU-CAU-GAU-CUC-AAA-AUG-AAA-CAG-ACG-UUU-CGC-UAU-AUC-AUC-UAC-AAG-AUU-GAU-GGU-GAC-AAU-AUU-GUC-ACU-GAC-UCU-GUU-GGA-AAU-CGA-GAG-GAA-ACG-UUU-GAG-AAU-UUC-AUC-AAA-GUC-AUU-CCU-CAA-AAU-AAU-GCU-CGU-UAU-GCU-GUG-UUU-GAC-UAC-UAC-UUC-AAG-ACC-AAU-GAA-GUU-CCU-UCA-AGG-GAA-AUU-CAG-AAA-CUG-AUA-UUC-AUU-UUC-UAC-UGU-CCU-GAC-ACU-GCU-CCU-CUC-AAA-GAA-AAG-AUG-CUC-UAU-GCU-UCU-UCC-AAA-GAU-GAU"
> ret$value.list  # The weight for each codon pairs.
[1] "(0)-(2)-(2)-(2)-(2)-(1)-(0)-(2)-(0)-(2)-(0)-(1)-(2)-(0)-(2)-(0)-(0)-(1)-(2)-(0)-(0)-(2)-(0)-(2)-(0)-(0)-(0)-(2)-(1)-(2)-(1)-(2)-(0)-(1)-(2)-(0)-(2)-(2)-(0)-(2)-(0)-(2)-(0)-(0)-(0)-(2)-(0)-(2)-(2)-(2)-(0)-(2)-(2)-(0)-(2)-(2)-(2)-(0)-(0)-(2)-(2)-(0)-(2)-(0)-(2)-(2)-(2)-(2)-(0)-(2)-(0)-(2)-(2)-(0)-(2)-(2)-(2)-(0)-(0)-(0)-(2)-(2)-(2)-(1)-(2)-(0)-(2)-(1)-(0)-(0)-(0)-(2)-(2)-(0)-(2)-(2)-(2)-(0)-(0)-(1)-(2)-(1)-(2)-(2)-(2)-(2)"
> ret$total   # The sum of the weight.
[1] 122
> ret$value.tab   # The distribution of weight.
value
 0  1  2
40 10 56
```
